# Significance of tumor mutation burden combined with immune infiltrates in the progression and prognosis of ovarian cancer

**DOI:** 10.1186/s12935-020-01472-9

**Published:** 2020-08-05

**Authors:** Fangfang Bi, Ying Chen, Qing Yang

**Affiliations:** 1 Department of Obstetrics and Gynecology, Shengjing Hospital of China Medical University, NO. 36 Sanhao Road, Shenyang, 110000 China; 2 Department of Ultrasound, Jiangnan Hospital Affiliated to Zhejiang University of Traditional Chinese Medicine, Hangzhou, China

**Keywords:** OC, TIICs, Prognosis, TMB, Maftools, CIBERSORT, CMap

## Abstract

**Background:**

Ovarian cancer (OC) is the most malignant tumor in the female reproductive system. About 75% of OC in complete remission of clinical symptoms still develop a recurrence. Therefore, searching for new treatment methods plays an important role in improving the prognosis of OC.

**Methods:**

We downloaded the MAF files, RNA-seq data and clinical information from the TCGA database. The “maftools” package in R software was used to visualize the OC mutation data. We calculated the tumor mutation burden (TMB) of OC and analyzed its correlation with clinicopathological parameters and prognostic value. Tumor mutation burden related signature model was constructed to predict the overall survival (OS) of OC.

**Results:**

The results revealed that there was a statistical correlation between TMB and FIGO stage, grade and tumor residual size of ovarian cancer patients. The Kaplan–Meier curve indicated that a high TMB is associated with better clinical outcomes of OC. The difference analysis indicated 24 upregulated genes and 619 downregulated genes in the high-TMB group compared with the low-TMB group. Besides, the TMBRS model based on five hub genes (RBMS3, PLA2G5, CDH2, AMHR2 and ADAMTS8) was constructed to predict the OS of OC. The ROC curve and validation data sets all revealed that the TMBRS model was reliable in predicting recurrence risk. Immune microenvironment analysis indicated the correlations between TMB and infiltrating immune cells.

**Conclusions:**

Our results suggest that TMB plays an important role in the prognosis and guiding immunotherapy of OC. By detecting the TMB of OC, clinicians can more accurately treat patients with immunotherapy, thereby improving their survival rate.

## Background

Ovarian cancer is one of the most malignant tumors in the female reproductive system and ranks second only to cervical cancer in global incidence and mortality [1]. Due to a lack of early symptoms and effective early screening diagnostic methods, most patients with OC are found in the late stage, and the 5-year survival rate is only 20–25% [2]. The main treatment method is a combination of tumor cell ablation and chemotherapy drugs, such as paclitaxel and platinum-based drugs. Despite the development of diagnostic and treatment technology, the mortality rate has not improved significantly [3]. Therefore, searching for new treatment methods plays an important role in improving the prognosis of patients with OC.

Immunotherapy is a kind of therapy that can enhance the autoimmune ability of patients to kill or eliminate cancer cells. Immunotherapy includes many methods, such as tumor vaccine [4], immunocytotherapy, therapeutic antibody, small synthetic molecule inhibitors, immune checkpoint inhibitors, etc. Among them, immunocheckpoint inhibitors play a very important role in tumor treatment. Immunocheckpoint inhibitors have been used in melanoma [5], non-small cell lung cancer [6], Hodgkin’s lymphoma [7] and many other tumors. In recent years, immunotherapy, as a new treatment of ovarian cancer, has gradually attracted people’s attention and achieved some results in the treatment of ovarian cancer. Especially the inhibitors for immunocheckpoint of PD1/PDL1. Unfortunately, the overall response rate of patients to these inhibitors is still low [8].

The tumor mutation burden (TMB) refers to the total number of replacement and insertion/deletion (indel) mutations per basic group in the exon coding region of the assessed gene in the genome of a tumor cell. Driver gene mutations can lead to the occurrence of tumors, but a large number of somatic mutations produce neoantigens, which activate CD8+ cytotoxic T cells and exert an anti-tumor effect mediated by T cells. Thus, more neoantigens are produced as the number of genetic variations increases, and the more likely it is that the immune system will recognize them. TMB was originally intended as a biomarker for predictive efficacy in patients with advanced melanoma treated with ipilimumab or tremelimumab. Patients with melanoma and a high TMB level tend to have better efficacy against PD-1/PD-L1 checkpoint inhibitors than patients with a low TMB level [9]. In recent years, treatment with PD-1/PD-L1 checkpoint inhibitors has developed rapidly, opening a new chapter in the treatment of advanced OC, but patients have shown low objective response rates [10]. Therefore, finding suitable biomarkers to screen the dominant population and improve the efficacy of immunotherapy is the top priority of immunotherapy for OC.

In this study, we calculated the TMB in 397 patients with OC in the TCGA database. Then, we investigated the relationship between TMB, prognosis, and clinicopathological parameters, such as grade, FIGO stage, lymphatic metastasis, and vascular invasion in patients with OC. Finally, we investigated the gene expression and tumor infiltrating immune cells (TIICs) related to TMB. After a comprehensive analysis of the TMB of OC cases in the TCGA database, we determined that TMB plays an important role in the malignant progression and prognosis of OC. Thus, monitoring patient mutation load can be used to provide more accurate immunotherapy.

## Materials and methods

### TCGA data acquisition

We downloaded the OC genetic mutation data, transcriptome data, and clinical data from the TCGA database (/) [11]. The genetic mutation data contained 37,248 mutated genes. The transcriptome data included 307 cases of OC. The clinical data included age, sex, grade, FIGO stage, lymph node metastasis, and vascular invasion. The gene microarray data and corresponding clinical information of verifying cohorts GSE9891 [12] and GSE26193 [13] was downloaded from GEO database (https://www.ncbi.nlm.nih.gov/geo/, RRID:SCR_007303). The data were standardized, and R software (R Foundation for Statistical Computing, Vienna, Austria, RRID:SCR_003302) was used for all operations.

### Calculation of TMB in patients with OC

TMB was defined as the number of somatic, coding, base replacement, and insert-deletion mutations per megabase of the genome examined using non-synonymous and code-shifting indels under a 5% detection limit. We used R software and the following formula to calculate the TMB of the patients with OC:

$$ {\text{TMB}} = {\text{Sn}} \times {{1,000,000} \mathord{\left/ {\vphantom {{1,000,000} {\text{n}}}} \right. \kern-0pt} {\text{n}}} $$

where Sn represents the absolute number of somatic mutations, and n represents the number of exon base coverage depth ≥ 100×) [14].

### Differential analysis and Functional enrichment analysis

Ovarian cancer data were divided into two groups according to median TMB value. Through the algorithm of limma package, the differentially expressed genes were calculated, and the genes with logFDR < 0.05 and lg|Fold change| (log|FC|) > 1 were selected as the significantly differentially expressed genes. In order to better understand the function of the selected differentially expressed genes, we use enrich GO in the clusterprofiler package of R to perform the GO function enrichment analysis and KEGG pathway enrichment analysis. The false discovery rate (FDR) was less than 0.01.

### Identification and verification of hub TMB‐related signature

The expression data and survival data of the selected differential genes were combined, and univariate Cox proportional hazards regression (PHR) analysis was performed to obtain survival-related genes. The genes with the *p* values (*p *< 0.001) were fitted in a multivariate Cox PHR model establish an risk score model. Kaplan–Meier survival curve was drawn to evaluate the difference of overall survival rate between high and low risk groups (p < 0.05). The receiver operating characteristic (ROC) curve was calculated to assess the predictive power of the risk score model. Finally, the result was test in verifying cohorts GSE9891 and GSE26193.

### Estimate of immune cell infiltration

CIBERSORT is a deconvolution algorithm that combines the labeled genomes of different immune cell subpopulations to calculate the proportion of 22 immune cells in tissues. The 22 types of immune cells included: 7 types of T cells (CD8+ T cells, naive CD4+ T cells, resting memory CD4+ T cells, activated memory CD4+ T cells, follicle-assisted T cells, regulatory T cells, and γδT cells), 3 types of B cells (naive B cells, memory B cells, and plasma cells) NK cells (resting NK cells and activated NK cells), and various myeloid cells (monocytes, M0 macrophages, M1 macrophages, M2 macrophages, resting dendritic cells, activated dendritic cells, resting mast cells, activated mast cells, eosinophils, and neutrophils). In this study, the CIBERSORT online platform (http://cibersort.stanford.edu/) was used to complete the calculation, and each sample was assigned a *p* value. Samples with a CIBERSORT output value of p < 0.05 were screened for further analysis [15].

### Identification of potential compounds

CMAP database stores up gene expression profile data of human cell lines including MCF7, ssMCF7, PC3, HL60 and SKMEL5 processed by 1309 bioactive small molecules. Differentially expressed genes based on TMB value were divided into up- and downregulated groups. The probe IDs of the two groups genes were uploaded to the connectivity map website (https://portals.broadinstitute.org/cmap/), respectively, and then a permuted results were obtained.

### Statistical analyses

SPSS 23.0 software (SPSS Inc., Chicago, IL, USA) was used for data recording and analysis, and the Kolmogorov–Smirnov test was used to determine whether variables obeyed a normal distribution. If the data were normally distributed, the mean ± standard deviation was calculated and the independent sample *t*-test was used to detect differences between groups. If a normal distribution was not observed, the median value was presented, and the non-parametric rank sum test was used to detect differences between the groups. Comparisons of classified data between the groups were analyzed by the Chi square test, and p < 0.05 was considered significant. The follow-up endpoint was overall survival (OS), which refers to the time from the date of the definite diagnosis of OC patients to death from any cause or the end of the final follow-up. The survival curve was plotted by Kaplan–Meier method, and the differences between the groups were assessed by the log-rank test. Cox proportional hazard model was used to evaluate the effect of clinical variables and TMB level on the OS of the patients with OC, and a p-value < 0.05 was considered significant.

## Results

### Somatic mutations in the OC data

**Fig. 1:**
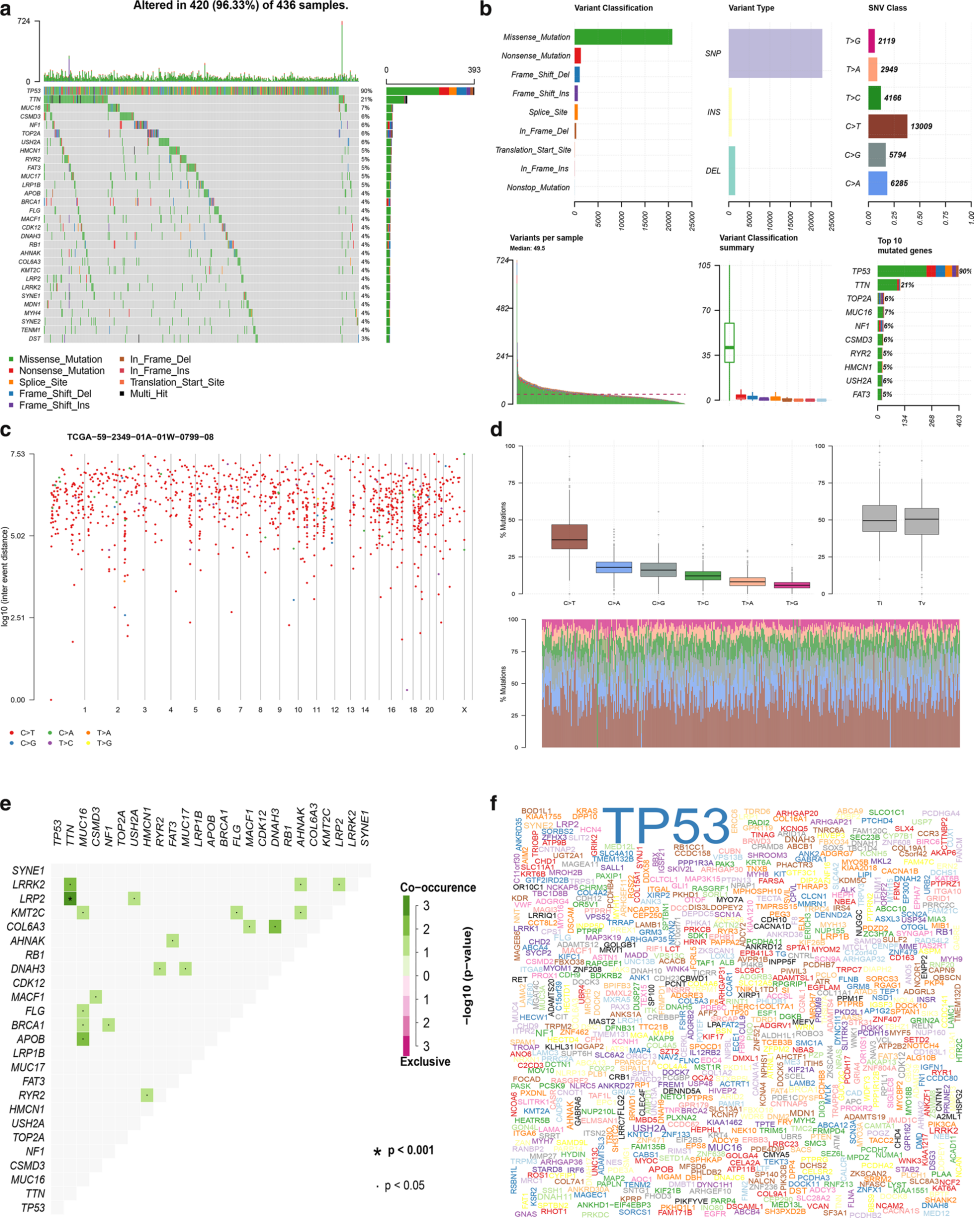
Genome‐wide mutation profiling in OC. **A** Landscape of mutation profiles in OC samples. Mutation information of each gene in each sample was shown in the waterfall plot, where different colors with specific annotations at the bottom meant the various mutation types. The barplot above the legend exhibited the number of mutation burden. **B** Cohort summary plot displaying distribution of variants according to variant classification, type and SNV class. Bottom part (from left to right) indicates mutation load for each sample, variant classification type. A stacked barplot shows top ten mutated genes. **C** Rainfall plot of TCGA ovarian cancer sample TCGA-59-2349-01A-01W-0799-08. Each point is a mutation color coded according to SNV class. **D** Transition and transversion plot displaying distribution of SNVs in OC classified into six transition and transversion events. Stacked bar plot (bottom) shows distribution of mutation spectra for every sample in the MAF file. **E** Mutually co-occurring gene pairs in OC displayed as a triangular matrix. **F** A word cloud generated based on frequency of mutated genes in OC

To identify the somatic mutations of the patients with OC in the TCGA database, mutation data were downloaded and visualized using the “maftools” package in R software. Horizontal histogram showed the genes have the higher mutation frequency in patients with OC, such as TP53 (90%), TTN (21%), MUC16 (7%), TOP2A (6%), and NF1(6%, Fig. 1A, Bf, F). Missense mutations were the most common type of mutation in patients with OC (Fig. 1Ba), single nucleotide polymorphism (SNP) occupied an absolute position compared with insertion (INS) or deletion (DEL, Fig. 1Bb), and C>T was the predominant mutation type detected (Fig. 1Bc, D). The number of mutations per sample was shown in Fig. 1Bd. In Fig. 1Be, the box diagram of each color represents a kind of mutation. Cancer genomes, especially solid tumors are characterized by genomic loci with localized hyper-mutations. Such hyper mutated genomic regions can be visualized by plotting inter variant distance on a linear genomic scale. These plots generally called rainfall plots. Figure 1C revealed the rainfall plot of TCGA ovarian cancer sample TCGA-59-2349-01A-01W-0799-08. Each point is a mutation color coded according to SNV class. Figure 1E shows that LRP2 and TTN have the highest correlation.

### Drug–gene interactions and oncogenic signaling pathways

**Fig. 2:**
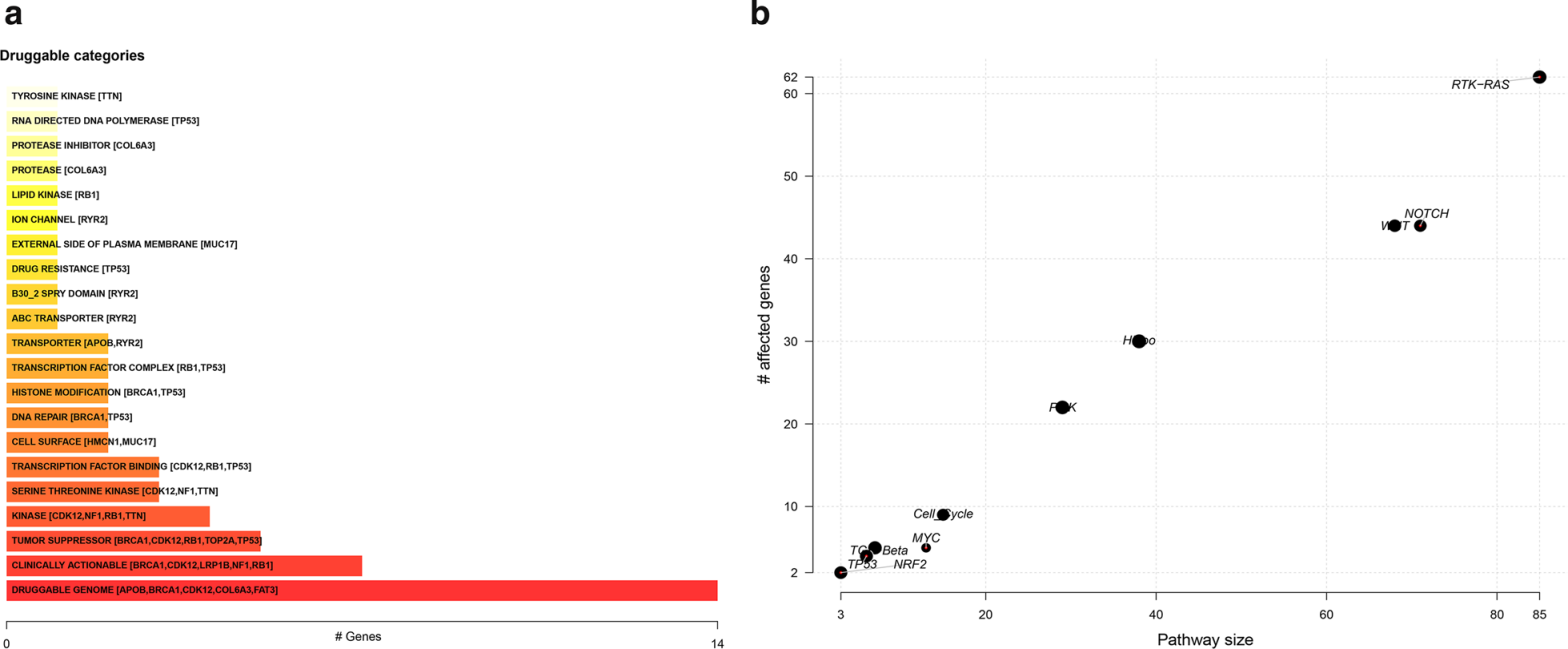
Drug–gene interactions and oncogenic signaling pathways. **a** Drug–gene interactions, potential druggable gene categories along with top 5 genes involved in them. **b** Oncogenic signaling pathways in TCGA cohorts and the genes affected by these pathways

From the professional definition, pharmacogenomics is to explore the influence of genetic variation of genes on the therapeutic effect of drugs from the perspective of genome. Drug–gene interaction database (DGIdb, http://dgidb.org/) is a database used to mine existing resources and generate information about how mutant genes are targeted or prioritized for drug development [16]. Figure 2a showed potential druggable gene categories along with top 5 genes involved in them. We could see that the drug group targeting TP53 is the largest and tumor suppressors targeting BRCA1, cdk12, RB1 genes have been developed to clinical application stage. Besides, we also discussed the enrichment of known Oncogenic Signaling Pathways in TCGA cohorts and the genes affected by these pathways (Fig. 2b). We could see the abundance of RTK-RAS oncogenic signaling pathway in ovarian cancer samples and the number of genes affected by this pathway is the highest.

### TMB was associated with survival outcomes, FIGO stage, tumor grade, and tumor residual size

**Fig. 3:**
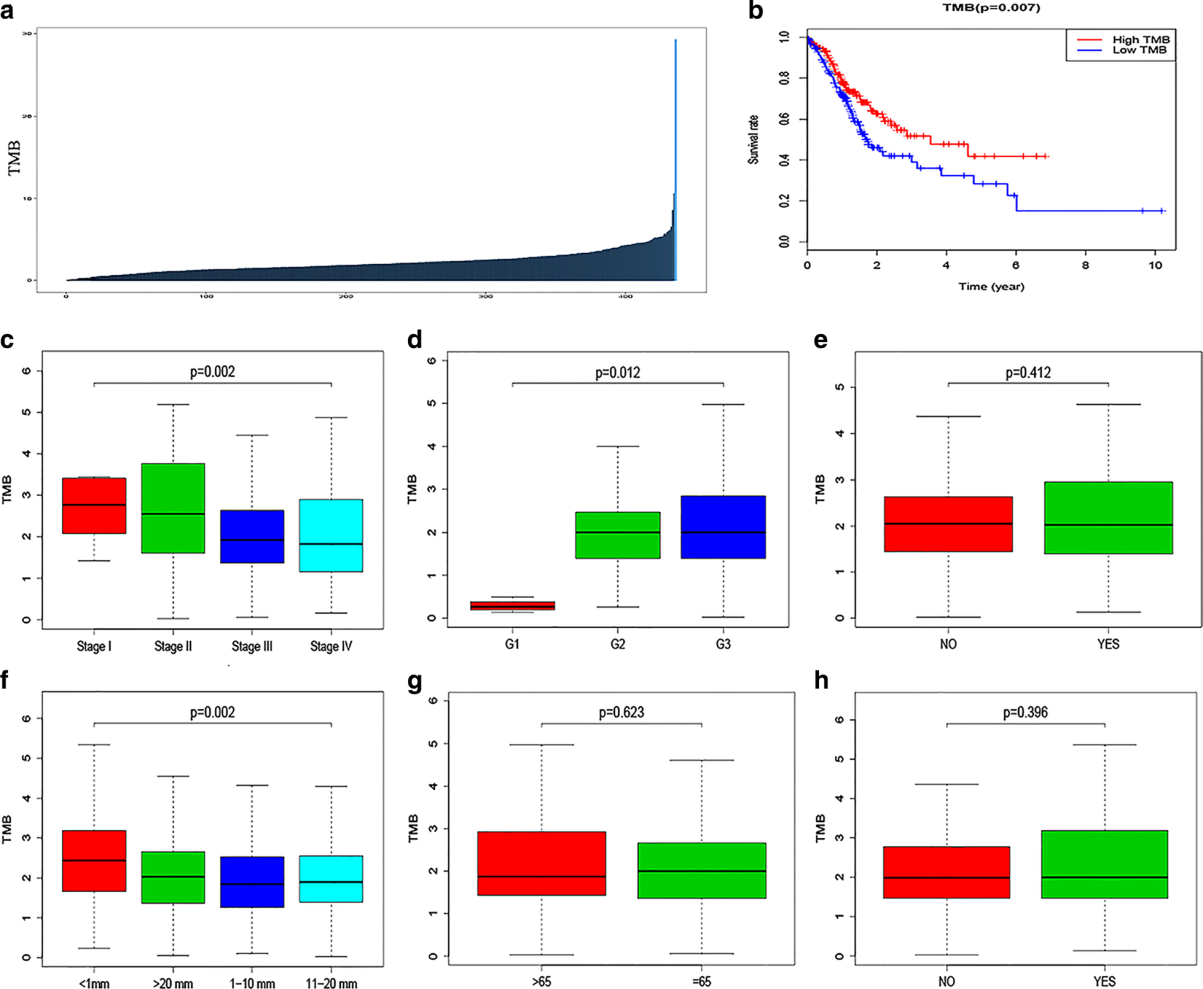
Prognostic analysis of TMB and associations with clinicopathological parameters. **a** The TMB value were showed by a box plot. **b** Kaplan–Meier curve indicated that a high TMB was associated with a better clinical outcome of patients with OC. **c**, **f** TMB is negatively correlated with FIGO stage (**c**) or tumor residual size (**f**) of OC. **d** TMB is positively correlated with the grade of OC. **e**, **g**, **h** No significant correlations between TMB and lymph node invasion (**e**), age (**g**) or vascular invasion (**h**)

TMB value of per TCGA ovarian cancer was calculated and revealed by Histogram (Fig. 3a). To investigate the correlation between TMB and the prognosis of patients with OC, we downloaded the prognostic information of the patients with OC and plotted a Kaplan–Meier curve. The results indicated that a high TMB was associated with a better clinical outcome of patients with OC (p = 0.007, Fig. 3b). Then, we downloaded the clinical information to investigate the correlation between TMB and the clinicopathological parameters of the patients with OC. We then mapped the correlation between TMB and the clinicopathological parameters, such as age, grade, FIGO stage, lymph node invasion, tumor residual size, and vascular invasion. The results revealed no significant correlations between TMB and lymph node invasion (p = 0.412, Fig. 3e), age (p = 0.623, Fig. 3g) or vascular invasion (p = 0.396, Fig. 3h). TMB is negatively correlated with FIGO stage (p = 0.002, Fig. 3c) or tumor residual size (p = 0.002, Fig. 3f) of ovarian cancer, while it is positively correlated with the grade (p = 0.012, Fig. 3d) of ovarian cancer.

### Genetic changes associated with TMB and functional analysis

**Fig. 4:**
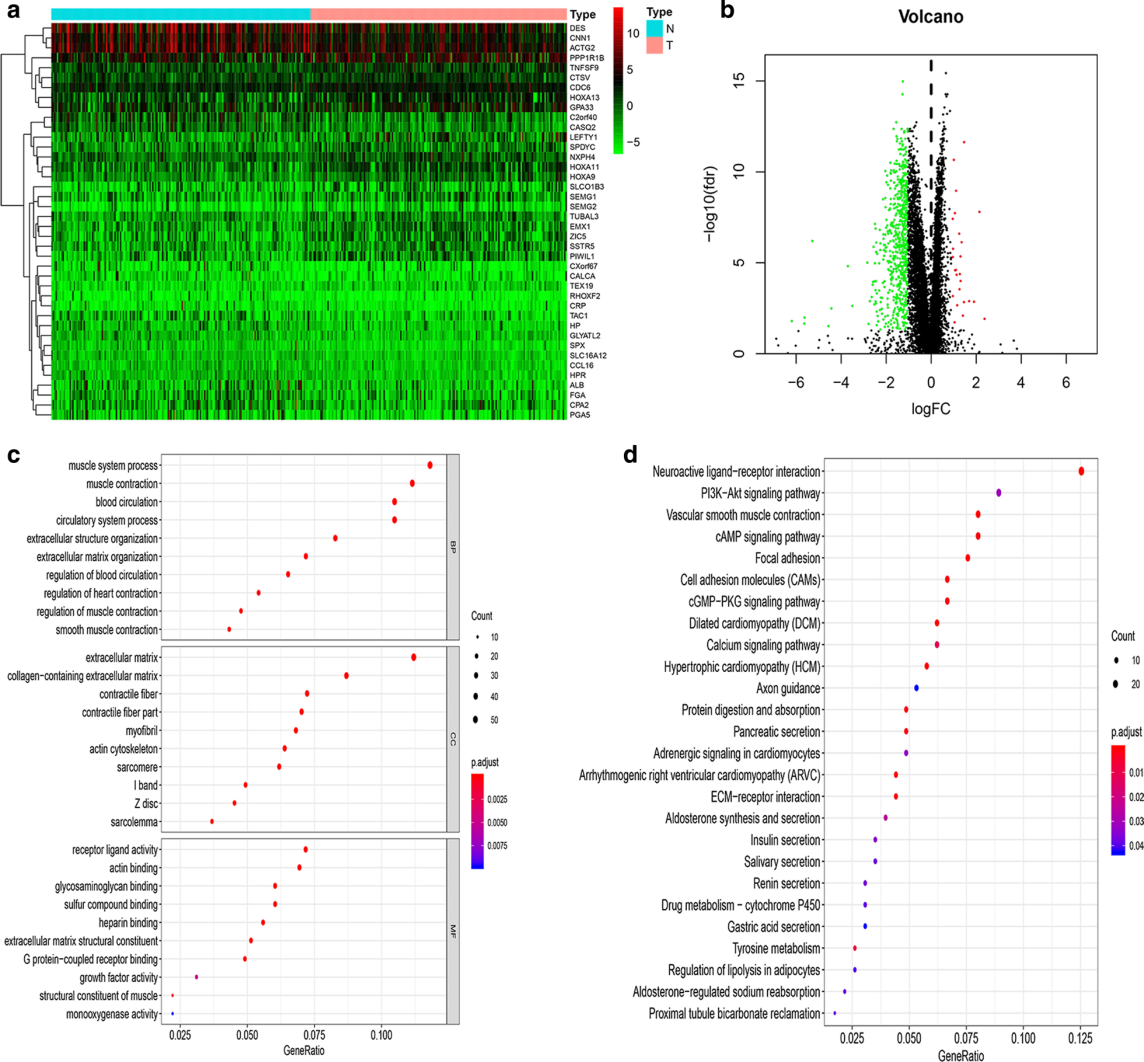
Genetic changes associated with TMB and functional analysis. **A** The heat map of top 40 differentially expressed genes. **B** Volcanic map showed the differentially expressed genes (DEGs) associated with TMB. Red dots are positive correlations, green dots are negative correlations. **C** GO functional enrichment analysis of the differentially expressed genes. **D** KEGG pathway functional enrichment analysis of the differentially expressed genes. Circle color represents p value, size represents gene number involved in

To investigate the differentially expressed genes (DEGs) associated with TMB in the OC cases, we divided the patients with OC into a high-TMB group and a low-TMB group. The edgeR package was used to screen DEGs between the high-TMB and low-TMB groups. The results indicated 24 upregulated genes and 619 downregulated genes in the high-TMB group compared with the low-TMB group. Figure 4a is a heat map of the TOP 40 differentially expressed genes, which indicated that he level of gene expression is generally decreased in the high TMB group. The DEGs in the high-TMB and low-TMB groups of patients with OC were visualized in a volcanic map (Fig. 4b). In GO functional analysis, muscle contraction, muscle system process, and extracellular matrix were enriched (Fig. 4c, Additional file 1: Table S1). In KEGG pathway analysis, the genes mainly enriched in Neuroactive ligand-receptor interaction, Calcium signaling pathway, and Vascular smooth muscle contraction (Fig. 4d, Additional file 2: Table S2).

### Identification and evaluation of 5 hub TMB‐related signature

**Fig. 5:**
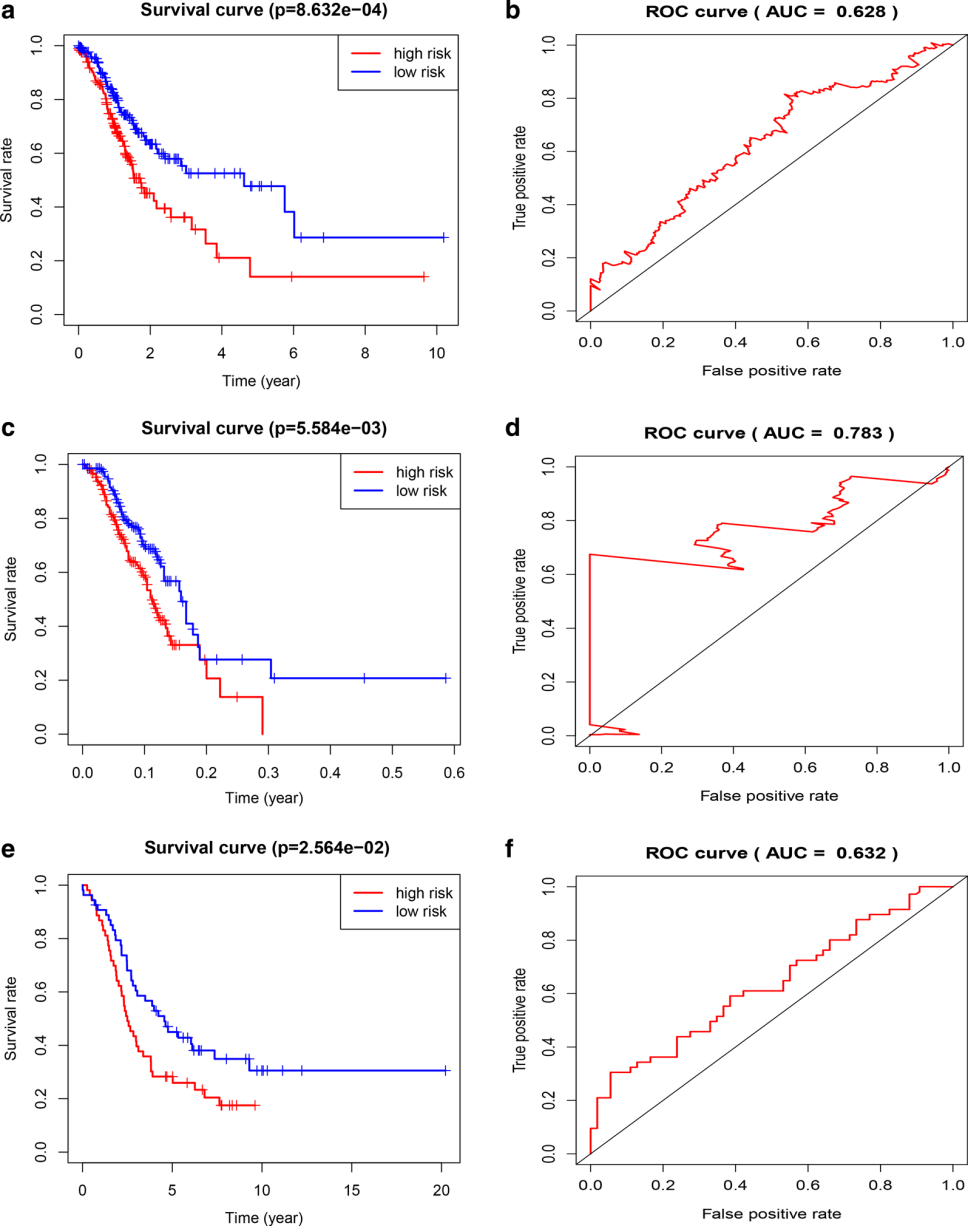
Establishment of TMBRS model. **a** K–M curves for TCGA cohort. **b** Time‐dependent ROC curves showed the predictive efficiency of the TMBRS model in TCGA cohort. **C** K–M curves for GSE9891 cohort. **D** Time‐dependent ROC curves showed the predictive efficiency of the TMBRS model in GSE9891 cohort. **e** K–M curves for GSE26193 cohort. **f** Time‐dependent ROC curves showed the predictive efficiency of the TMBRS model in GSE26193 cohort

We utilized univariate Cox PHR to screened 17 survival related genes among the differentially expressed genes (Additional file 3: Table S3), and performed multivariate Cox PHR to further selected 5 genes to establish TMBRS model. The estimated regression coefficients are as follows:

$$ Risk_{5} = 0.16x_{1} + 0.05x_{2} + 0.05x_{3} + 0.17x_{4} + 0.11x_{5} $$

where *x*_1_ represents the expression of AMHR2, *x*_2_ represents CDH2, *x*_3_ represents ADAMTS8, *x*_4_ represents RBMS3, and *x*_5_ represents PLA2G5. The patients were divided into two groups according to the median value of risk score, and patients in high-risk group have poor outcome (Fig. 5a). The ROC curve revealed that the TMBRS model was reliable in predicting recurrence risk (Fig. 5b). Then, the results were validated in other datasets GSE9891 (Fig. 5c, d), GSE26193 (Fig. 5e, f).

### Tumor infiltrating immune cells (TIICs) associated with TMB

**Fig. 6:**
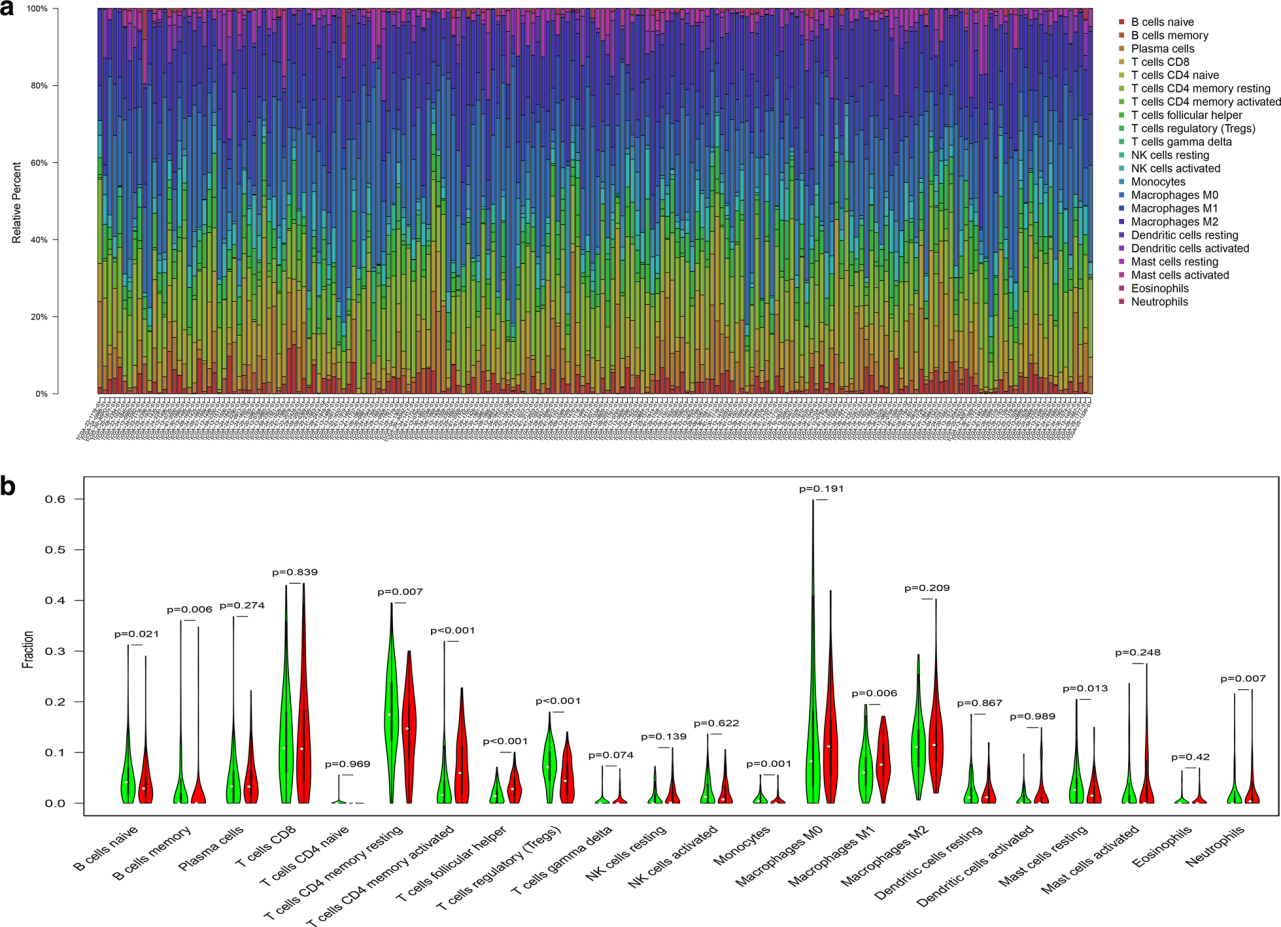
TIICs associated with TMB. **a** The landscape of TIICs in TCGA cohort. **b** TIICs associated with TMB. Red means high TMB, green means low TMB

To investigate the correlation between TIICs and TMB in OC, we first used CIBERSORT to calculate infiltration of 22 immune cells in the OC cases (Fig. 6a). We found that macrophages account for the largest proportion among 22 immune cells. Then, we divided the OC cases into high-TMB and low-TMB groups according to the frequency of TMB. The difference analytical results showed that naive B cells, memory B cells, resting memory CD4+ T cells, Tregs, monocytes, resting mast cells, and neutrophils were higher infiltrating in low‐TMB group, while activated memory CD4+ T cells, follicle-assisted T cells, M1 macrophages were higher infiltrating in high‐TMB group (Fig. 6b). Recent studies have reported that activated memory CD4+ T cells, follicle-assisted T cells and M1 macrophages play a crucial role in antitumor immunity [17–19]. We speculated from the results that high TMB can induce the activation of antitumor immune cells in OC patients and improve the prognosis of OC patients, while low TMB can’ t.

### Connectivity map analysis identifies candidate compounds

Differentially expressed genes were divided into up- and downregulated groups, the gene ID of which were converted to probe ID and uploaded to the connectivity map website. Among the highly correlated compounds, ciclopirox, thiethylperazine, cefepime and tetrandrine showed higher positive correlation with TMB of OC, which might improve OS of patients with a higher TMB (Table 1).

**Table 1:** Highly positive correlated compounds with TMB of OC obtained from connectivity map website

Rank	Cmap name	Mean	n	Enrichment	p
1	Ciclopirox	0.703	4	0.91	0.00006
2	Thiethylperazine	0.561	4	0.711	0.01436
3	Cefepime	0.544	4	0.789	0.00394
4	Tetrandrine	0.529	4	0.815	0.00219
5	Prestwick-967	0.506	4	0.849	0.00074
6	Karakoline	0.468	6	0.592	0.01502
7	Terazosin	0.467	4	0.659	0.03093
8	Memantine	0.465	4	0.761	0.00621
9	Ethisterone	0.443	6	0.562	0.0261
10	Thiamazole	0.411	6	0.55	0.03141
11	Cinnarizine	0.396	4	0.672	0.0255
12	Sulfacetamide	0.329	4	0.712	0.014
13	Alclometasone	0.323	4	0.671	0.02572
14	Altizide	0.291	4	0.7	0.01671

## Discussion

OC is a highly malignant tumor that seriously threatens a woman’s health. There are no typical clinical symptoms and signs in its early stage. Once symptoms appear, most cases are in an advanced stage, and the mortality rate is the highest among gynecological malignant tumors [20]. Although cytoreductive surgery and platinum-based combination chemotherapy have improved the 5-year survival rate of patients with OC, there has been no substantial progress in clinical diagnosis and treatment of OC [21]. Therefore, finding new treatments is crucial to improve the survival rate of patients with OC.

Gene mutations are changes in the molecular structure of genes caused by the replacement, addition, or deletion of DNA base pairs. According to the way genetic information changes, gene mutations can be divided into three types: same sense mutations, missense mutations, and nonsense mutations [22]. Same sense mutations do not have an actual mutation effect, while missense and nonsense mutations in most cases affect the structure and function of proteins or enzymes, thereby changing the genetic information. In our study, the mutations in the patients with OC were mainly missense mutations. The distribution of mutation sites in the gene is different, most of which occur on some mutation hot spots [23]. Therefore, it is of great significance for diagnosis and treatment of tumor-related diseases to search for these hot mutated genes by gene sequencing technology. In our research, we found that TP53 had a high mutation frequency in patients with OC.

TMB is an important biological marker reflecting the degree of tumor mutation. Alexandrov and Lawrence et al. found that the TMB among tumor samples was significantly different, which was at least 0.001/Mb and up to 400/Mb. The TMB of different patients is also significantly different even for the same type of tumor. Some studies have reported that the TMB as a biological marker has an important correlation with the therapeutic effect of cancer immunotherapy [24]. The reason why TMB is a marker of immunotherapy stems from the biological mechanism of somatic mutation and the immune response. Somatic mutations of tumors include synonymous mutations and non-synonymous mutations. Non-synonymous mutations produce abnormal proteins by changing the amino acid sequence. However, the immunogenicity of abnormal proteins in tumors is the basis of the tumor immune response. If abnormal proteins are finally recognized by immune cells, they will become neoantigens, and subsequent immune responses can develop [25]. That is to say, when the TMB of a tumor sample is high, the mutations that produce immunogenic neoantigens in the mutations also increase. It is easier for the immune system to recognize and remove tumor cells, and the survival rate of patients will be relatively improved. In our study, the OS of the patients with OC in the high TMB group was significantly higher than that in the low TMB group, which was consistent with previous assumptions. However, We were unable to validate our predictions in other OC datasets due to the lack of prognostic information. In addition, we found that there was a statistical correlation between TMB and FIGO stages, Grade or tumor residual size. Then, five genes (RBMS3, PLA2G5, CDH2, AMHR2 and ADAMTS8) were selected to establish TMBRS model based on univariate and multivariate Cox PHR. The ROC curve and validation data sets all revealed that the TMBRS model was reliable in predicting recurrence risk. However, further we need more clinical trials to verify the results.

TIICs are part of the tumor microenvironment that promote, regulate, and inhibit the development and growth of tumors. According to the interactions between the types and functions of immune cells, immune cells may play a variety of roles in the development of tumors [26]. In our study, we used the CIBERSORT algorithm to calculate the proportion of 22 immune cells in OC. The patients with OC were divided into two groups according to the TMB naive B cells, memory B cells, resting memory CD4+ T cells, Tregs, monocytes, resting mast cells, and neutrophils were higher infiltrating in low‐TMB group, while activated memory CD4+ T cells, follicle-assisted T cells, M1 macrophages were higher infiltrating in high‐TMB group, which indirectly confirms the previous view that a high TMB of tumors can induce the immune response of the body and thus inhibit the growth of tumors.

## Conclusions

In conclusion, our results suggest that TMB, as an important biomarker of tumor mutation, plays an important role in the prognosis and guiding immunotherapy of OC. By determining the TMB of patients with OC, clinicians can more accurately treat patients with immunotherapy, thereby improving their survival rate.

## Supplementary information

**Additional file 1: Table S1.** GO functional enrichment analysis of the differentially expressed genes.

**Additional file 2: Table S2.** KEGG pathway functional enrichment analysis of the differentially expressed genes.

**Additional file 3: Table S3.** Univariate Cox PHR of the differentially expressed genes.

## Data Availability

All data generated or analysed during this study are included in this published article and its additional files

## Abbreviations

OC: Ovarian cancer
TMBRS: Tumor mutation burden related signature
OS: Overall survival
TIICs: Tumor infiltrating immune cells
TMB: Tumor mutation burden
FDR: False discovery rate
PHR: Proportional hazards regression
ROC): Receiver operating characteristic
SNP: single nucleotide polymorphism
INS: Insertion
DEL: Deletion
DEGs: Differentially expressed genes
