# Supplementary material for: Significance of tumor mutation burden combined with immune infiltrates in the progression and prognosis of ovarian cancer
Source: Cancer Cell Int. 2020 Aug 5;20:373. doi: 10.1186/s12935-020-01472-9 (PMC7405355; doi:10.1186/s12935-020-01472-9)
Supplement: Supplementary file 1 — Additional file 1: Table S1. GO functional enrichment analysis of the differentially expressed genes. [file 12935_2020_1472_MOESM1_ESM.docx]

| Description | pvalue | p.adjust | qvalue | gene count |
| --- | --- | --- | --- | --- |
| muscle contraction | 7.34E-26 | 2.97E-22 | 2.39E-22 | 57 |
| muscle system process | 1.22E-23 | 2.46E-20 | 1.98E-20 | 62 |
| regulation of blood circulation | 1.93E-15 | 2.60E-12 | 2.09E-12 | 40 |
| heart contraction | 1.04E-12 | 1.05E-09 | 8.48E-10 | 35 |
| regulation of heart contraction | 1.52E-12 | 1.22E-09 | 9.86E-10 | 33 |
| heart process | 2.68E-12 | 1.81E-09 | 1.45E-09 | 35 |
| regulation of membrane potential | 6.99E-12 | 4.03E-09 | 3.25E-09 | 40 |
| multicellular organismal signaling | 2.55E-10 | 1.29E-07 | 1.04E-07 | 26 |
| actin-mediated cell contraction | 3.14E-10 | 1.41E-07 | 1.13E-07 | 20 |
| smooth muscle contraction | 4.32E-10 | 1.74E-07 | 1.40E-07 | 19 |
| cellular metal ion homeostasis | 5.13E-10 | 1.83E-07 | 1.48E-07 | 42 |
| cardiac conduction | 5.45E-10 | 1.83E-07 | 1.48E-07 | 22 |
| calcium ion homeostasis | 1.15E-09 | 3.57E-07 | 2.88E-07 | 37 |
| regulation of muscle contraction | 1.25E-09 | 3.61E-07 | 2.90E-07 | 23 |
| regulation of cytosolic calcium ion concentration | 1.60E-09 | 3.89E-07 | 3.13E-07 | 31 |
| cellular calcium ion homeostasis | 1.62E-09 | 3.89E-07 | 3.13E-07 | 36 |
| modulation of chemical synaptic transmission | 1.64E-09 | 3.89E-07 | 3.13E-07 | 37 |
| regulation of trans-synaptic signaling | 1.76E-09 | 3.94E-07 | 3.17E-07 | 37 |
| extracellular structure organization | 3.77E-09 | 7.79E-07 | 6.28E-07 | 36 |
| actin filament-based movement | 3.86E-09 | 7.79E-07 | 6.28E-07 | 20 |
| regulation of muscle system process | 4.16E-09 | 8.00E-07 | 6.44E-07 | 28 |
| cellular divalent inorganic cation homeostasis | 5.31E-09 | 9.74E-07 | 7.84E-07 | 36 |
| divalent inorganic cation homeostasis | 5.92E-09 | 9.97E-07 | 8.03E-07 | 37 |
| positive regulation of cytosolic calcium ion concentration | 5.92E-09 | 9.97E-07 | 8.03E-07 | 28 |
| action potential | 6.82E-09 | 1.10E-06 | 8.87E-07 | 19 |
| phospholipase C-activating G protein-coupled receptor signaling pathway | 2.28E-08 | 3.55E-06 | 2.86E-06 | 14 |
| second-messenger-mediated signaling | 2.44E-08 | 3.65E-06 | 2.94E-06 | 33 |
| muscle organ development | 3.59E-08 | 5.17E-06 | 4.16E-06 | 35 |
| extracellular matrix organization | 4.91E-08 | 6.84E-06 | 5.50E-06 | 31 |
| cell-substrate adhesion | 7.44E-08 | 1.00E-05 | 8.06E-06 | 30 |
| cardiac muscle cell contraction | 1.28E-07 | 1.66E-05 | 1.34E-05 | 13 |
| positive regulation of ion transport | 2.18E-07 | 2.75E-05 | 2.21E-05 | 26 |
| membrane repolarization | 2.66E-07 | 3.25E-05 | 2.62E-05 | 11 |
| striated muscle contraction | 2.92E-07 | 3.46E-05 | 2.79E-05 | 20 |
| regulation of heart rate | 3.00E-07 | 3.46E-05 | 2.79E-05 | 15 |
| regulation of metal ion transport | 3.58E-07 | 4.02E-05 | 3.23E-05 | 31 |
| ossification | 4.47E-07 | 4.88E-05 | 3.93E-05 | 32 |
| morphogenesis of a branching structure | 5.08E-07 | 5.40E-05 | 4.35E-05 | 21 |
| potassium ion transport | 6.04E-07 | 6.22E-05 | 5.01E-05 | 21 |
| morphogenesis of a branching epithelium | 6.16E-07 | 6.22E-05 | 5.01E-05 | 20 |
| muscle cell differentiation | 7.79E-07 | 7.67E-05 | 6.18E-05 | 30 |
| mesenchymal cell proliferation | 8.45E-07 | 8.13E-05 | 6.55E-05 | 10 |
| cell communication involved in cardiac conduction | 8.96E-07 | 8.42E-05 | 6.78E-05 | 11 |
| sodium ion homeostasis | 1.05E-06 | 9.64E-05 | 7.77E-05 | 10 |
| regulation of membrane repolarization | 1.33E-06 | 0.000119 | 9.62E-05 | 9 |
| cellular potassium ion transport | 1.45E-06 | 0.000124 | 9.96E-05 | 17 |
| potassium ion transmembrane transport | 1.45E-06 | 0.000124 | 9.96E-05 | 17 |
| calcium-mediated signaling | 1.47E-06 | 0.000124 | 9.96E-05 | 20 |
| calcium ion transmembrane import into cytosol | 1.62E-06 | 0.000134 | 0.000108 | 16 |
| release of sequestered calcium ion into cytosol | 1.73E-06 | 0.000137 | 0.000111 | 15 |
| negative regulation of sequestering of calcium ion | 1.73E-06 | 0.000137 | 0.000111 | 15 |
| muscle cell development | 2.01E-06 | 0.000156 | 0.000125 | 18 |
| regulation of cardiac conduction | 2.12E-06 | 0.000161 | 0.00013 | 12 |
| regulation of sequestering of calcium ion | 2.18E-06 | 0.000163 | 0.000131 | 15 |
| calcium ion transport into cytosol | 2.37E-06 | 0.000173 | 0.00014 | 17 |
| vascular process in circulatory system | 2.40E-06 | 0.000173 | 0.00014 | 18 |
| cardiac muscle cell action potential | 2.46E-06 | 0.000174 | 0.00014 | 12 |
| G protein-coupled receptor signaling pathway, coupled to cyclic nucleotide second messenger | 2.55E-06 | 0.000178 | 0.000143 | 19 |
| cardiac muscle contraction | 3.02E-06 | 0.000204 | 0.000164 | 16 |
| sequestering of calcium ion | 3.03E-06 | 0.000204 | 0.000164 | 15 |
| regulation of release of sequestered calcium ion into cytosol by sarcoplasmic reticulum | 3.22E-06 | 0.000213 | 0.000171 | 8 |
| regulation of cell-substrate adhesion | 4.14E-06 | 0.000268 | 0.000215 | 20 |
| cell-cell signaling involved in cardiac conduction | 4.17E-06 | 0.000268 | 0.000215 | 8 |
| regulation of action potential | 5.88E-06 | 0.000371 | 0.000299 | 10 |
| axon development | 6.00E-06 | 0.000373 | 0.0003 | 32 |
| cardiac muscle cell action potential involved in contraction | 6.96E-06 | 0.000418 | 0.000337 | 10 |
| positive regulation of cell-substrate adhesion | 7.03E-06 | 0.000418 | 0.000337 | 14 |
| cytosolic calcium ion transport | 7.05E-06 | 0.000418 | 0.000337 | 17 |
| regulation of actin filament-based process | 7.57E-06 | 0.000443 | 0.000357 | 27 |
| transmembrane receptor protein serine/threonine kinase signaling pathway | 8.32E-06 | 0.00048 | 0.000386 | 26 |
| release of sequestered calcium ion into cytosol by sarcoplasmic reticulum | 8.61E-06 | 0.00049 | 0.000394 | 8 |
| cognition | 9.21E-06 | 0.000509 | 0.00041 | 24 |
| maintenance of location | 9.21E-06 | 0.000509 | 0.00041 | 24 |
| regulation of potassium ion transport | 9.42E-06 | 0.000514 | 0.000414 | 13 |
| negative chemotaxis | 9.92E-06 | 0.000527 | 0.000424 | 7 |
| export across plasma membrane | 9.92E-06 | 0.000527 | 0.000424 | 7 |
| striated muscle cell development | 1.03E-05 | 0.000538 | 0.000433 | 16 |
| response to alcohol | 1.06E-05 | 0.000544 | 0.000438 | 21 |
| regulation of heart rate by cardiac conduction | 1.08E-05 | 0.000544 | 0.000438 | 8 |
| release of sequestered calcium ion into cytosol by endoplasmic reticulum | 1.08E-05 | 0.000544 | 0.000438 | 8 |
| branching morphogenesis of an epithelial tube | 1.12E-05 | 0.000558 | 0.00045 | 16 |
| divalent metal ion transport | 1.25E-05 | 0.000609 | 0.000491 | 32 |
| signal release | 1.25E-05 | 0.000609 | 0.000491 | 31 |
| muscle tissue development | 1.29E-05 | 0.000613 | 0.000494 | 29 |
| sensory perception of pain | 1.32E-05 | 0.000613 | 0.000494 | 13 |
| regulation of hormone secretion | 1.32E-05 | 0.000613 | 0.000494 | 22 |
| regulation of smooth muscle contraction | 1.32E-05 | 0.000613 | 0.000494 | 10 |
| cartilage development | 1.35E-05 | 0.000621 | 0.0005 | 18 |
| calcium ion transmembrane transport | 1.38E-05 | 0.000626 | 0.000504 | 24 |
| calcium ion transport | 1.44E-05 | 0.000644 | 0.000519 | 30 |
| divalent inorganic cation transport | 1.48E-05 | 0.000659 | 0.000531 | 32 |
| regulation of cellular response to growth factor stimulus | 1.58E-05 | 0.000692 | 0.000557 | 23 |
| mesonephros development | 1.63E-05 | 0.000708 | 0.00057 | 13 |
| regulation of anatomical structure size | 1.87E-05 | 0.000797 | 0.000641 | 31 |
| regulation of blood pressure | 1.87E-05 | 0.000797 | 0.000641 | 17 |
| hormone secretion | 2.15E-05 | 0.000902 | 0.000726 | 24 |
| atrial cardiac muscle cell action potential | 2.21E-05 | 0.000902 | 0.000726 | 6 |
| atrial cardiac muscle cell to AV node cell signaling | 2.21E-05 | 0.000902 | 0.000726 | 6 |
| atrial cardiac muscle cell to AV node cell communication | 2.21E-05 | 0.000902 | 0.000726 | 6 |
| monovalent inorganic cation homeostasis | 2.34E-05 | 0.000943 | 0.000759 | 14 |
| learning or memory | 2.72E-05 | 0.001068 | 0.00086 | 21 |
| bone mineralization | 2.73E-05 | 0.001068 | 0.00086 | 13 |
| response to prostaglandin | 2.76E-05 | 0.001068 | 0.00086 | 7 |
| regulation of cardiac muscle cell membrane repolarization | 2.76E-05 | 0.001068 | 0.00086 | 7 |
| axon guidance | 2.78E-05 | 0.001068 | 0.00086 | 19 |
| sarcoplasmic reticulum calcium ion transport | 2.97E-05 | 0.001132 | 0.000911 | 8 |
| regulation of tube diameter | 3.08E-05 | 0.00115 | 0.000926 | 14 |
| regulation of blood vessel diameter | 3.08E-05 | 0.00115 | 0.000926 | 14 |
| neuron projection guidance | 3.17E-05 | 0.001173 | 0.000945 | 19 |
| positive regulation of animal organ morphogenesis | 3.24E-05 | 0.00118 | 0.00095 | 11 |
| learning | 3.24E-05 | 0.00118 | 0.00095 | 15 |
| monovalent inorganic cation transport | 3.31E-05 | 0.001192 | 0.00096 | 32 |
| forebrain development | 3.41E-05 | 0.001218 | 0.000981 | 27 |
| cell-matrix adhesion | 3.60E-05 | 0.001277 | 0.001028 | 19 |
| hormone transport | 3.65E-05 | 0.001283 | 0.001033 | 24 |
| synapse organization | 3.74E-05 | 0.001303 | 0.001049 | 28 |
| regulation of transmembrane receptor protein serine/threonine kinase signaling pathway | 3.84E-05 | 0.001317 | 0.00106 | 19 |
| regulation of cation transmembrane transport | 3.85E-05 | 0.001317 | 0.00106 | 24 |
| adenylate cyclase-modulating G protein-coupled receptor signaling pathway | 4.11E-05 | 0.001393 | 0.001122 | 16 |
| striated muscle tissue development | 4.30E-05 | 0.001446 | 0.001164 | 27 |
| regulation of ossification | 4.50E-05 | 0.00149 | 0.0012 | 18 |
| positive regulation of transmembrane transport | 4.50E-05 | 0.00149 | 0.0012 | 18 |
| positive regulation of potassium ion transport | 5.08E-05 | 0.001668 | 0.001344 | 8 |
| regulation of calcium ion transmembrane transporter activity | 5.14E-05 | 0.001674 | 0.001348 | 11 |
| semaphorin-plexin signaling pathway | 5.32E-05 | 0.001712 | 0.001379 | 6 |
| developmental induction | 5.34E-05 | 0.001712 | 0.001379 | 7 |
| regulation of morphogenesis of a branching structure | 5.57E-05 | 0.001771 | 0.001426 | 9 |
| regulation of blood vessel size | 6.14E-05 | 0.001936 | 0.001559 | 14 |
| connective tissue development | 6.64E-05 | 0.00207 | 0.001667 | 20 |
| regulation of tube size | 6.66E-05 | 0.00207 | 0.001667 | 14 |
| striated muscle cell differentiation | 6.81E-05 | 0.0021 | 0.001691 | 21 |
| regulation of cardiac muscle contraction by regulation of the release of sequestered calcium ion | 6.91E-05 | 0.002115 | 0.001703 | 6 |
| regulation of ion transmembrane transport | 7.33E-05 | 0.002226 | 0.001793 | 30 |
| gliogenesis | 7.61E-05 | 0.002294 | 0.001847 | 22 |
| biomineral tissue development | 8.31E-05 | 0.002487 | 0.002002 | 15 |
| skeletal system development | 8.53E-05 | 0.002532 | 0.002039 | 31 |
| regulation of ryanodine-sensitive calcium-release channel activity | 8.86E-05 | 0.002608 | 0.0021 | 6 |
| potassium ion homeostasis | 8.91E-05 | 0.002608 | 0.0021 | 5 |
| skeletal muscle tissue development | 9.63E-05 | 0.002797 | 0.002252 | 15 |
| glial cell differentiation | 0.000102 | 0.002951 | 0.002376 | 18 |
| positive regulation of cation transmembrane transport | 0.000107 | 0.003073 | 0.002475 | 14 |
| membrane depolarization during action potential | 0.000112 | 0.003192 | 0.00257 | 6 |
| positive regulation of potassium ion transmembrane transport | 0.000116 | 0.003268 | 0.002632 | 7 |
| regulation of ion transmembrane transporter activity | 0.00012 | 0.003364 | 0.002709 | 19 |
| potassium ion import across plasma membrane | 0.000123 | 0.003428 | 0.00276 | 5 |
| lung morphogenesis | 0.000131 | 0.003622 | 0.002916 | 8 |
| regulation of release of sequestered calcium ion into cytosol | 0.000134 | 0.00369 | 0.002972 | 10 |
| excretion | 0.000141 | 0.003845 | 0.003096 | 9 |
| negative regulation of blood vessel diameter | 0.00015 | 0.004056 | 0.003266 | 10 |
| chemokine-mediated signaling pathway | 0.000151 | 0.00407 | 0.003277 | 8 |
| positive regulation of synaptic transmission | 0.000156 | 0.004176 | 0.003363 | 14 |
| actomyosin structure organization | 0.000158 | 0.004185 | 0.00337 | 15 |
| SA node cell to atrial cardiac muscle cell communication | 0.00016 | 0.004234 | 0.003409 | 4 |
| muscle filament sliding | 0.000164 | 0.004253 | 0.003424 | 7 |
| actin-myosin filament sliding | 0.000164 | 0.004253 | 0.003424 | 7 |
| synaptic transmission, cholinergic | 0.000166 | 0.004253 | 0.003424 | 5 |
| potassium ion import | 0.000166 | 0.004253 | 0.003424 | 5 |
| endocrine process | 0.000166 | 0.004253 | 0.003424 | 10 |
| memory | 0.000172 | 0.00436 | 0.003511 | 12 |
| regulation of cardiac muscle contraction by calcium ion signaling | 0.000174 | 0.004366 | 0.003515 | 6 |
| regulation of osteoblast proliferation | 0.000174 | 0.004366 | 0.003515 | 6 |
| regulation of striated muscle contraction | 0.000175 | 0.004366 | 0.003515 | 11 |
| neuropeptide signaling pathway | 0.000179 | 0.00444 | 0.003576 | 9 |
| skeletal muscle organ development | 0.00018 | 0.00444 | 0.003576 | 15 |
| osteoblast differentiation | 0.000183 | 0.004477 | 0.003605 | 18 |
| regulation of transmembrane transporter activity | 0.000186 | 0.004519 | 0.003639 | 19 |
| regulation of vasoconstriction | 0.000199 | 0.004821 | 0.003882 | 8 |
| multi-organism behavior | 0.000202 | 0.004847 | 0.003903 | 9 |
| ureteric bud development | 0.000211 | 0.005002 | 0.004028 | 11 |
| regulation of calcium ion transport into cytosol | 0.000211 | 0.005002 | 0.004028 | 11 |
| epithelial tube branching involved in lung morphogenesis | 0.000214 | 0.005056 | 0.004071 | 6 |
| multi-multicellular organism process | 0.000216 | 0.005069 | 0.004082 | 18 |
| digestion | 0.000218 | 0.005069 | 0.004082 | 13 |
| digestive tract development | 0.000218 | 0.005069 | 0.004082 | 13 |
| mesonephric epithelium development | 0.00023 | 0.005287 | 0.004258 | 11 |
| mesonephric tubule development | 0.00023 | 0.005287 | 0.004258 | 11 |
| positive regulation of vascular permeability | 0.000246 | 0.005611 | 0.004519 | 4 |
| regulation of potassium ion transmembrane transport | 0.00025 | 0.005673 | 0.004568 | 10 |
| positive regulation of ion transmembrane transport | 0.000256 | 0.00578 | 0.004654 | 14 |
| regulation of neutrophil chemotaxis | 0.000261 | 0.005849 | 0.00471 | 6 |
| regulation of cardiac muscle cell contraction | 0.000266 | 0.005891 | 0.004744 | 7 |
| cardiac muscle cell membrane repolarization | 0.000266 | 0.005891 | 0.004744 | 7 |
| response to ketone | 0.000285 | 0.006289 | 0.005064 | 16 |
| substrate adhesion-dependent cell spreading | 0.000304 | 0.006663 | 0.005365 | 10 |
| regulation of endocrine process | 0.000309 | 0.006742 | 0.005429 | 7 |
| positive regulation of sodium ion transport | 0.000315 | 0.006767 | 0.005449 | 6 |
| osteoblast proliferation | 0.000315 | 0.006767 | 0.005449 | 6 |
| vasoconstriction | 0.000315 | 0.006767 | 0.005449 | 9 |
| regulation of sodium ion transport | 0.00035 | 0.007486 | 0.006028 | 9 |
| negative regulation of transmembrane receptor protein serine/threonine kinase signaling pathway | 0.000354 | 0.007532 | 0.006065 | 12 |
| regulation of granulocyte chemotaxis | 0.000358 | 0.007532 | 0.006065 | 7 |
| keratan sulfate catabolic process | 0.00036 | 0.007532 | 0.006065 | 4 |
| AV node cell to bundle of His cell communication | 0.00036 | 0.007532 | 0.006065 | 4 |
| regulation of actin cytoskeleton organization | 0.000367 | 0.007629 | 0.006143 | 21 |
| activation of adenylate cyclase activity | 0.000378 | 0.007822 | 0.006298 | 6 |
| regulation of transporter activity | 0.000398 | 0.008195 | 0.006599 | 19 |
| regulation of neurotransmitter levels | 0.000412 | 0.008439 | 0.006795 | 22 |
| mesenchyme development | 0.000417 | 0.008474 | 0.006824 | 19 |
| negative regulation of response to external stimulus | 0.000418 | 0.008474 | 0.006824 | 23 |
| positive regulation of epithelial cell proliferation | 0.000451 | 0.0091 | 0.007328 | 16 |
| organ induction | 0.000461 | 0.009174 | 0.007387 | 5 |
| response to prostaglandin E | 0.000461 | 0.009174 | 0.007387 | 5 |
| regulation of renal sodium excretion | 0.000461 | 0.009174 | 0.007387 | 5 |
| endocrine hormone secretion | 0.000474 | 0.009232 | 0.007434 | 7 |
| response to chemokine | 0.000477 | 0.009232 | 0.007434 | 8 |
| cellular response to chemokine | 0.000477 | 0.009232 | 0.007434 | 8 |
| positive regulation of hormone secretion | 0.000478 | 0.009232 | 0.007434 | 12 |
| cAMP-mediated signaling | 0.000478 | 0.009232 | 0.007434 | 13 |
| digestive system development | 0.000478 | 0.009232 | 0.007434 | 13 |
| cellular component assembly involved in morphogenesis | 0.000481 | 0.009242 | 0.007442 | 10 |
| actin filament organization | 0.000487 | 0.009321 | 0.007505 | 22 |
| developmental maturation | 0.000505 | 0.009613 | 0.007741 | 19 |
| axonogenesis | 0.000522 | 0.0099 | 0.007972 | 25 |
| fat cell differentiation | 0.000531 | 0.009948 | 0.00801 | 17 |
| regulation of mesenchymal cell proliferation | 0.000532 | 0.009948 | 0.00801 | 6 |
| regulation of neutrophil migration | 0.000532 | 0.009948 | 0.00801 | 6 |
| regulation of actin filament-based movement | 0.000542 | 0.010089 | 0.008124 | 7 |
| membrane depolarization during cardiac muscle cell action potential | 0.000574 | 0.010641 | 0.008569 | 5 |
| platelet degranulation | 0.000592 | 0.010915 | 0.008789 | 12 |
| axon regeneration | 0.000618 | 0.011266 | 0.009072 | 7 |
| integrin-mediated signaling pathway | 0.000623 | 0.011266 | 0.009072 | 10 |
| positive regulation of ossification | 0.000623 | 0.011266 | 0.009072 | 10 |
| oligodendrocyte differentiation | 0.000623 | 0.011266 | 0.009072 | 10 |
| cyclic-nucleotide-mediated signaling | 0.000628 | 0.011266 | 0.009072 | 14 |
| regulation of cation channel activity | 0.000628 | 0.011266 | 0.009072 | 14 |
| positive regulation of chemotaxis | 0.00068 | 0.012157 | 0.009789 | 12 |
| response to inactivity | 0.000693 | 0.01233 | 0.009929 | 4 |
| receptor clustering | 0.000702 | 0.012421 | 0.010002 | 7 |
| regulation of inflammatory response to antigenic stimulus | 0.000707 | 0.012421 | 0.010002 | 5 |
| renal sodium excretion | 0.000707 | 0.012421 | 0.010002 | 5 |
| renal system development | 0.000717 | 0.012527 | 0.010087 | 20 |
| response to xenobiotic stimulus | 0.000727 | 0.012625 | 0.010166 | 19 |
| regulation of animal organ formation | 0.000732 | 0.012625 | 0.010166 | 6 |
| regulation of digestive system process | 0.000732 | 0.012625 | 0.010166 | 6 |
| lung development | 0.00075 | 0.012881 | 0.010372 | 14 |
| sodium ion transport | 0.000769 | 0.013156 | 0.010594 | 15 |
| positive regulation of response to external stimulus | 0.000789 | 0.013451 | 0.010831 | 21 |
| regulation of cardiac muscle contraction | 0.000842 | 0.01421 | 0.011443 | 9 |
| postsynaptic membrane organization | 0.000851 | 0.01421 | 0.011443 | 6 |
| positive regulation of calcium ion transmembrane transporter activity | 0.000851 | 0.01421 | 0.011443 | 6 |
| neuron migration | 0.000851 | 0.01421 | 0.011443 | 13 |
| negative regulation of cell development | 0.000856 | 0.01421 | 0.011443 | 21 |
| cell morphogenesis involved in neuron differentiation | 0.00086 | 0.01421 | 0.011443 | 29 |
| regulation of excretion | 0.000862 | 0.01421 | 0.011443 | 5 |
| regulation of ventricular cardiac muscle cell membrane repolarization | 0.000862 | 0.01421 | 0.011443 | 5 |
| protein polymerization | 0.000872 | 0.014313 | 0.011525 | 17 |
| cell chemotaxis | 0.000876 | 0.014325 | 0.011535 | 18 |
| BMP signaling pathway | 0.000891 | 0.014447 | 0.011634 | 12 |
| regulation of smooth muscle cell proliferation | 0.000891 | 0.014447 | 0.011634 | 14 |
| myofibril assembly | 0.000898 | 0.014502 | 0.011678 | 7 |
| cellular response to transforming growth factor beta stimulus | 0.000914 | 0.014663 | 0.011808 | 17 |
| neuron cell-cell adhesion | 0.000922 | 0.014663 | 0.011808 | 4 |
| potassium ion export | 0.000922 | 0.014663 | 0.011808 | 4 |
| potassium ion export across plasma membrane | 0.000922 | 0.014663 | 0.011808 | 4 |
| respiratory tube development | 0.000943 | 0.014928 | 0.012021 | 14 |
| respiratory system development | 0.000953 | 0.015028 | 0.012102 | 15 |
| negative regulation of potassium ion transport | 0.000984 | 0.015407 | 0.012406 | 6 |
| ventricular cardiac muscle cell action potential | 0.000984 | 0.015407 | 0.012406 | 6 |
| smooth muscle cell proliferation | 0.000997 | 0.015544 | 0.012517 | 14 |
| regulation of multicellular organism growth | 0.001008 | 0.015651 | 0.012603 | 8 |
| positive regulation of ion transmembrane transporter activity | 0.001013 | 0.015665 | 0.012614 | 10 |
| hormone metabolic process | 0.001039 | 0.016021 | 0.012901 | 16 |
| adult behavior | 0.001082 | 0.016607 | 0.013373 | 12 |
| urogenital system development | 0.001127 | 0.017237 | 0.01388 | 21 |
| response to transforming growth factor beta | 0.001209 | 0.018419 | 0.014832 | 17 |
| postsynapse organization | 0.001217 | 0.018451 | 0.014857 | 13 |
| peripheral nervous system development | 0.001225 | 0.018451 | 0.014857 | 8 |
| positive regulation of cation channel activity | 0.001225 | 0.018451 | 0.014857 | 8 |
| chemical synaptic transmission, postsynaptic | 0.001272 | 0.019088 | 0.015371 | 10 |
| lung alveolus development | 0.001299 | 0.019434 | 0.015649 | 6 |
| kidney epithelium development | 0.001306 | 0.019453 | 0.015664 | 12 |
| response to antibiotic | 0.001313 | 0.019489 | 0.015694 | 21 |
| neurotransmitter transport | 0.001323 | 0.019572 | 0.01576 | 17 |
| neuron projection regeneration | 0.001416 | 0.020868 | 0.016804 | 7 |
| regulation of amine transport | 0.001477 | 0.021471 | 0.017289 | 8 |
| protein-containing complex remodeling | 0.001478 | 0.021471 | 0.017289 | 5 |
| protein-lipid complex remodeling | 0.001478 | 0.021471 | 0.017289 | 5 |
| plasma lipoprotein particle remodeling | 0.001478 | 0.021471 | 0.017289 | 5 |
| ERK1 and ERK2 cascade | 0.001618 | 0.023418 | 0.018857 | 19 |
| regulation of calcium ion transmembrane transport | 0.001662 | 0.023974 | 0.019305 | 12 |
| epithelial tube morphogenesis | 0.001734 | 0.024915 | 0.020062 | 20 |
| cell communication by electrical coupling | 0.001742 | 0.024937 | 0.020081 | 5 |
| positive regulation of transporter activity | 0.001822 | 0.025992 | 0.02093 | 10 |
| regulation of chemotaxis | 0.001882 | 0.0266 | 0.021419 | 14 |
| response to ethanol | 0.001884 | 0.0266 | 0.021419 | 11 |
| regulation of postsynaptic membrane potential | 0.001884 | 0.0266 | 0.021419 | 11 |
| regulation of ERK1 and ERK2 cascade | 0.001897 | 0.026665 | 0.021471 | 18 |
| response to nicotine | 0.00191 | 0.026665 | 0.021471 | 6 |
| regulation of actin filament organization | 0.001911 | 0.026665 | 0.021471 | 16 |
| embryonic digestive tract morphogenesis | 0.001922 | 0.026665 | 0.021471 | 4 |
| vocalization behavior | 0.001922 | 0.026665 | 0.021471 | 4 |
| regulation of animal organ morphogenesis | 0.001958 | 0.027079 | 0.021806 | 17 |
| response to BMP | 0.00198 | 0.02719 | 0.021895 | 12 |
| cellular response to BMP stimulus | 0.00198 | 0.02719 | 0.021895 | 12 |
| regulation of embryonic development | 0.002005 | 0.027451 | 0.022105 | 11 |
| leukocyte chemotaxis | 0.002079 | 0.028367 | 0.022842 | 14 |
| response to axon injury | 0.002105 | 0.028619 | 0.023045 | 8 |
| protein localization to cell surface | 0.002142 | 0.02903 | 0.023376 | 7 |
| amine transport | 0.002291 | 0.030936 | 0.024911 | 8 |
| kidney development | 0.002315 | 0.031164 | 0.025095 | 18 |
| branching involved in ureteric bud morphogenesis | 0.002362 | 0.031467 | 0.025339 | 7 |
| neutrophil chemotaxis | 0.002362 | 0.031467 | 0.025339 | 7 |
| positive regulation of smooth muscle contraction | 0.002369 | 0.031467 | 0.025339 | 5 |
| ventricular cardiac muscle cell membrane repolarization | 0.002369 | 0.031467 | 0.025339 | 5 |
| cerebral cortex development | 0.002388 | 0.031617 | 0.025459 | 10 |
| digestive system process | 0.002416 | 0.031865 | 0.025659 | 9 |
| regulation of actin filament depolymerization | 0.002423 | 0.031865 | 0.025659 | 6 |
| excitatory postsynaptic potential | 0.002598 | 0.03386 | 0.027266 | 9 |
| heterotypic cell-cell adhesion | 0.002599 | 0.03386 | 0.027266 | 7 |
| synapse assembly | 0.002599 | 0.03386 | 0.027266 | 13 |
| cell junction assembly | 0.002641 | 0.034288 | 0.02761 | 15 |
| regulation of developmental growth | 0.002684 | 0.034729 | 0.027966 | 20 |
| primary alcohol metabolic process | 0.002701 | 0.034729 | 0.027966 | 8 |
| cardiac muscle cell development | 0.002701 | 0.034729 | 0.027966 | 8 |
| negative regulation of BMP signaling pathway | 0.002715 | 0.034803 | 0.028025 | 6 |
| positive regulation of phosphatidylinositol 3-kinase activity | 0.002738 | 0.034984 | 0.028171 | 5 |
| locomotory behavior | 0.002776 | 0.035363 | 0.028476 | 14 |
| cell-substrate junction assembly | 0.00279 | 0.03543 | 0.02853 | 9 |
| synaptic transmission, glutamatergic | 0.002854 | 0.035903 | 0.02891 | 7 |
| neural nucleus development | 0.002854 | 0.035903 | 0.02891 | 7 |
| regulation of potassium ion transmembrane transporter activity | 0.002854 | 0.035903 | 0.02891 | 7 |
| copulation | 0.002898 | 0.036116 | 0.029082 | 4 |
| positive regulation of glycoprotein biosynthetic process | 0.002898 | 0.036116 | 0.029082 | 4 |
| phasic smooth muscle contraction | 0.002898 | 0.036116 | 0.029082 | 4 |
| establishment of T cell polarity | 0.002955 | 0.036161 | 0.029118 | 3 |
| response to muscle inactivity involved in regulation of muscle adaptation | 0.002955 | 0.036161 | 0.029118 | 3 |
| response to denervation involved in regulation of muscle adaptation | 0.002955 | 0.036161 | 0.029118 | 3 |
| sodium ion export across plasma membrane | 0.002955 | 0.036161 | 0.029118 | 3 |
| positive regulation of digestive system process | 0.002955 | 0.036161 | 0.029118 | 3 |
| lung secretory cell differentiation | 0.002955 | 0.036161 | 0.029118 | 3 |
| gland development | 0.002999 | 0.036589 | 0.029463 | 24 |
| stem cell proliferation | 0.003088 | 0.037558 | 0.030243 | 10 |
| specification of animal organ identity | 0.003146 | 0.038152 | 0.030722 | 5 |
| regulation of response to drug | 0.003166 | 0.038272 | 0.030818 | 8 |
| regulation of calcium-mediated signaling | 0.003209 | 0.038682 | 0.031149 | 9 |
| actin filament bundle assembly | 0.003224 | 0.03875 | 0.031203 | 11 |
| peptide hormone secretion | 0.003324 | 0.039691 | 0.031961 | 16 |
| actin filament depolymerization | 0.003378 | 0.039691 | 0.031961 | 6 |
| digestive tract morphogenesis | 0.003378 | 0.039691 | 0.031961 | 6 |
| positive regulation of phospholipid metabolic process | 0.003378 | 0.039691 | 0.031961 | 6 |
| reproductive structure development | 0.003379 | 0.039691 | 0.031961 | 24 |
| positive regulation of MAPK cascade | 0.003393 | 0.039691 | 0.031961 | 27 |
| actin filament bundle organization | 0.003411 | 0.039691 | 0.031961 | 11 |
| cell-substrate adherens junction assembly | 0.00342 | 0.039691 | 0.031961 | 8 |
| ammonium transport | 0.00342 | 0.039691 | 0.031961 | 8 |
| focal adhesion assembly | 0.00342 | 0.039691 | 0.031961 | 8 |
| membrane depolarization | 0.00342 | 0.039691 | 0.031961 | 8 |
| maternal process involved in female pregnancy | 0.003421 | 0.039691 | 0.031961 | 7 |
| ethanol metabolic process | 0.003494 | 0.040192 | 0.032364 | 4 |
| sympathetic nervous system development | 0.003494 | 0.040192 | 0.032364 | 4 |
| cellular response to prostaglandin stimulus | 0.003494 | 0.040192 | 0.032364 | 4 |
| response to isoquinoline alkaloid | 0.003597 | 0.041029 | 0.033038 | 5 |
| response to morphine | 0.003597 | 0.041029 | 0.033038 | 5 |
| lung epithelium development | 0.003597 | 0.041029 | 0.033038 | 5 |
| positive regulation of cell morphogenesis involved in differentiation | 0.003607 | 0.041029 | 0.033038 | 11 |
| reproductive system development | 0.00369 | 0.041858 | 0.033706 | 24 |
| regulation of calcium ion transport | 0.00375 | 0.042194 | 0.033976 | 16 |
| multicellular organismal movement | 0.003751 | 0.042194 | 0.033976 | 6 |
| musculoskeletal movement | 0.003751 | 0.042194 | 0.033976 | 6 |
| regulation of supramolecular fiber organization | 0.00393 | 0.043287 | 0.034857 | 19 |
| renal system process | 0.003942 | 0.043287 | 0.034857 | 10 |
| establishment of lymphocyte polarity | 0.003971 | 0.043287 | 0.034857 | 3 |
| regulation of dendritic cell antigen processing and presentation | 0.003971 | 0.043287 | 0.034857 | 3 |
| gastro-intestinal system smooth muscle contraction | 0.003971 | 0.043287 | 0.034857 | 3 |
| response to muscle inactivity | 0.003971 | 0.043287 | 0.034857 | 3 |
| AV node cell action potential | 0.003971 | 0.043287 | 0.034857 | 3 |
| AV node cell to bundle of His cell signaling | 0.003971 | 0.043287 | 0.034857 | 3 |
| dendritic cell apoptotic process | 0.003971 | 0.043287 | 0.034857 | 3 |
| atrial cardiac muscle cell membrane repolarization | 0.003971 | 0.043287 | 0.034857 | 3 |
| regulation of dendritic cell apoptotic process | 0.003971 | 0.043287 | 0.034857 | 3 |
| regulation of systemic arterial blood pressure | 0.003977 | 0.043287 | 0.034857 | 8 |
| telencephalon development | 0.004058 | 0.044051 | 0.035471 | 16 |
| ureteric bud morphogenesis | 0.004069 | 0.044051 | 0.035472 | 7 |
| social behavior | 0.004154 | 0.044615 | 0.035926 | 6 |
| intraspecies interaction between organisms | 0.004154 | 0.044615 | 0.035926 | 6 |
| regulation of synaptic transmission, glutamatergic | 0.004154 | 0.044615 | 0.035926 | 6 |
| cardiac cell development | 0.004281 | 0.045727 | 0.036821 | 8 |
| granulocyte chemotaxis | 0.004281 | 0.045727 | 0.036821 | 8 |
| mesonephric tubule morphogenesis | 0.004426 | 0.047159 | 0.037974 | 7 |
| mesenchyme morphogenesis | 0.004589 | 0.04823 | 0.038837 | 6 |
| regulation of substrate adhesion-dependent cell spreading | 0.004589 | 0.04823 | 0.038837 | 6 |
| regulation of sodium ion transmembrane transport | 0.004589 | 0.04823 | 0.038837 | 6 |
| neuron recognition | 0.004634 | 0.04823 | 0.038837 | 5 |
| cyclic nucleotide metabolic process | 0.004634 | 0.04823 | 0.038837 | 5 |
| regulation of vascular permeability | 0.004634 | 0.04823 | 0.038837 | 5 |
| positive regulation of lipid kinase activity | 0.004634 | 0.04823 | 0.038837 | 5 |
| regulation of renal system process | 0.004634 | 0.04823 | 0.038837 | 5 |
| positive regulation of substrate adhesion-dependent cell spreading | 0.004634 | 0.04823 | 0.038837 | 5 |
| extracellular matrix | 2.80E-21 | 1.06E-18 | 8.05E-19 | 60 |
| collagen-containing extracellular matrix | 1.36E-19 | 2.56E-17 | 1.95E-17 | 53 |
| contractile fiber | 4.98E-19 | 6.26E-17 | 4.77E-17 | 38 |
| contractile fiber part | 1.51E-18 | 1.42E-16 | 1.09E-16 | 36 |
| myofibril | 3.64E-18 | 2.74E-16 | 2.09E-16 | 36 |
| sarcomere | 1.71E-15 | 1.07E-13 | 8.18E-14 | 31 |
| I band | 8.77E-14 | 4.72E-12 | 3.60E-12 | 24 |
| Z disc | 8.93E-13 | 4.21E-11 | 3.21E-11 | 22 |
| sarcolemma | 2.00E-10 | 8.36E-09 | 6.37E-09 | 21 |
| intercalated disc | 2.50E-08 | 9.41E-07 | 7.17E-07 | 12 |
| plasma membrane raft | 1.37E-07 | 4.68E-06 | 3.57E-06 | 16 |
| cell-cell contact zone | 1.71E-07 | 5.37E-06 | 4.09E-06 | 13 |
| neuronal cell body | 1.96E-07 | 5.68E-06 | 4.33E-06 | 36 |
| sarcoplasm | 4.65E-07 | 1.25E-05 | 9.54E-06 | 13 |
| sarcoplasmic reticulum | 7.68E-07 | 1.93E-05 | 1.47E-05 | 12 |
| actin cytoskeleton | 1.46E-06 | 3.43E-05 | 2.62E-05 | 33 |
| perikaryon | 1.67E-06 | 3.71E-05 | 2.83E-05 | 16 |
| membrane raft | 3.38E-06 | 6.97E-05 | 5.32E-05 | 26 |
| membrane microdomain | 3.59E-06 | 6.97E-05 | 5.32E-05 | 26 |
| plasma membrane protein complex | 3.70E-06 | 6.97E-05 | 5.32E-05 | 33 |
| caveola | 4.70E-06 | 8.42E-05 | 6.42E-05 | 12 |
| synaptic membrane | 4.91E-06 | 8.42E-05 | 6.42E-05 | 30 |
| membrane region | 6.74E-06 | 0.000111 | 8.43E-05 | 26 |
| costamere | 1.08E-05 | 0.000169 | 0.000129 | 6 |
| blood microparticle | 1.14E-05 | 0.000172 | 0.000131 | 16 |
| postsynaptic membrane | 1.76E-05 | 0.000255 | 0.000195 | 24 |
| cell-cell junction | 8.06E-05 | 0.001125 | 0.000857 | 28 |
| sarcoplasmic reticulum membrane | 9.28E-05 | 0.00125 | 0.000953 | 7 |
| basement membrane | 0.000102 | 0.001332 | 0.001015 | 11 |
| T-tubule | 0.000145 | 0.001824 | 0.00139 | 8 |
| intrinsic component of presynaptic membrane | 0.000216 | 0.002626 | 0.002001 | 10 |
| transmembrane transporter complex | 0.000268 | 0.003161 | 0.00241 | 20 |
| anchored component of membrane | 0.000316 | 0.003608 | 0.00275 | 14 |
| transporter complex | 0.00034 | 0.003774 | 0.002877 | 20 |
| actin filament bundle | 0.000408 | 0.004323 | 0.003295 | 8 |
| integral component of presynaptic membrane | 0.000413 | 0.004323 | 0.003295 | 9 |
| endoplasmic reticulum lumen | 0.000504 | 0.005136 | 0.003915 | 21 |
| anchored component of external side of plasma membrane | 0.000678 | 0.006543 | 0.004988 | 4 |
| cation-transporting ATPase complex | 0.000678 | 0.006543 | 0.004988 | 4 |
| adherens junction | 0.000694 | 0.006543 | 0.004988 | 29 |
| cell-substrate junction | 0.000717 | 0.006592 | 0.005025 | 25 |
| actomyosin | 0.00097 | 0.008704 | 0.006635 | 8 |
| presynapse | 0.001039 | 0.009105 | 0.00694 | 26 |
| dystrophin-associated glycoprotein complex | 0.001173 | 0.00983 | 0.007493 | 4 |
| glycoprotein complex | 0.001173 | 0.00983 | 0.007493 | 4 |
| focal adhesion | 0.00125 | 0.010241 | 0.007807 | 24 |
| presynaptic membrane | 0.00132 | 0.010502 | 0.008005 | 12 |
| cell-substrate adherens junction | 0.001337 | 0.010502 | 0.008005 | 24 |
| cation channel complex | 0.001452 | 0.011172 | 0.008516 | 14 |
| stress fiber | 0.001523 | 0.011258 | 0.008581 | 7 |
| contractile actin filament bundle | 0.001523 | 0.011258 | 0.008581 | 7 |
| neuron to neuron synapse | 0.001746 | 0.01266 | 0.00965 | 20 |
| asymmetric synapse | 0.001843 | 0.012938 | 0.009862 | 19 |
| platelet alpha granule | 0.001853 | 0.012938 | 0.009862 | 9 |
| neuromuscular junction | 0.002284 | 0.015368 | 0.011714 | 7 |
| A band | 0.002307 | 0.015368 | 0.011714 | 5 |
| intrinsic component of external side of plasma membrane | 0.002323 | 0.015368 | 0.011714 | 4 |
| M band | 0.002835 | 0.017661 | 0.013462 | 4 |
| endocytic vesicle lumen | 0.002835 | 0.017661 | 0.013462 | 4 |
| ATPase dependent transmembrane transport complex | 0.002835 | 0.017661 | 0.013462 | 4 |
| glutamatergic synapse | 0.00287 | 0.017661 | 0.013462 | 21 |
| fascia adherens | 0.002904 | 0.017661 | 0.013462 | 3 |
| external side of plasma membrane | 0.003316 | 0.019841 | 0.015124 | 14 |
| postsynaptic density | 0.003685 | 0.021709 | 0.016548 | 18 |
| interstitial matrix | 0.003903 | 0.021964 | 0.016742 | 3 |
| sodium:potassium-exchanging ATPase complex | 0.003903 | 0.021964 | 0.016742 | 3 |
| symmetric synapse | 0.003903 | 0.021964 | 0.016742 | 3 |
| myosin filament | 0.004078 | 0.02261 | 0.017234 | 4 |
| platelet alpha granule lumen | 0.004284 | 0.023161 | 0.017655 | 7 |
| ion channel complex | 0.004311 | 0.023161 | 0.017655 | 16 |
| apical part of cell | 0.004362 | 0.023161 | 0.017655 | 21 |
| intrinsic component of synaptic membrane | 0.004626 | 0.02422 | 0.018462 | 12 |
| apical plasma membrane | 0.005229 | 0.027003 | 0.020583 | 18 |
| sodium channel complex | 0.006465 | 0.032935 | 0.025105 | 3 |
| integral component of synaptic membrane | 0.007102 | 0.0357 | 0.027212 | 11 |
| postsynaptic specialization | 0.008768 | 0.043495 | 0.033154 | 18 |
| heparin binding | 7.31E-13 | 4.26E-10 | 3.60E-10 | 26 |
| extracellular matrix structural constituent | 2.23E-11 | 4.91E-09 | 4.14E-09 | 25 |
| glycosaminoglycan binding | 2.53E-11 | 4.91E-09 | 4.14E-09 | 28 |
| sulfur compound binding | 6.29E-11 | 9.17E-09 | 7.73E-09 | 29 |
| receptor ligand activity | 3.50E-08 | 4.08E-06 | 3.44E-06 | 35 |
| receptor regulator activity | 6.15E-08 | 5.98E-06 | 5.04E-06 | 36 |
| actin binding | 3.84E-07 | 3.20E-05 | 2.69E-05 | 31 |
| G protein-coupled peptide receptor activity | 9.02E-07 | 6.57E-05 | 5.54E-05 | 15 |
| peptide receptor activity | 1.02E-06 | 6.61E-05 | 5.58E-05 | 15 |
| structural constituent of muscle | 1.17E-06 | 6.83E-05 | 5.76E-05 | 9 |
| growth factor activity | 3.49E-06 | 0.000185 | 0.000156 | 17 |
| actin filament binding | 1.03E-05 | 0.000501 | 0.000423 | 16 |
| cation channel activity | 1.65E-05 | 0.00074 | 0.000624 | 22 |
| metal ion transmembrane transporter activity | 2.25E-05 | 0.000937 | 0.00079 | 26 |
| G protein-coupled receptor binding | 2.43E-05 | 0.000943 | 0.000795 | 19 |
| ion channel activity | 4.14E-05 | 0.001431 | 0.001207 | 25 |
| hormone binding | 4.17E-05 | 0.001431 | 0.001207 | 11 |
| substrate-specific channel activity | 6.43E-05 | 0.002083 | 0.001756 | 25 |
| ion gated channel activity | 0.000104 | 0.00297 | 0.002504 | 21 |
| channel activity | 0.000107 | 0.00297 | 0.002504 | 26 |
| gated channel activity | 0.000109 | 0.00297 | 0.002504 | 21 |
| passive transmembrane transporter activity | 0.000112 | 0.00297 | 0.002504 | 26 |
| inorganic cation transmembrane transporter activity | 0.000157 | 0.003982 | 0.003357 | 29 |
| steroid binding | 0.000255 | 0.0062 | 0.005227 | 10 |
| cation transmembrane transporter activity | 0.0003 | 0.006993 | 0.005896 | 30 |
| voltage-gated ion channel activity | 0.000443 | 0.009558 | 0.008059 | 14 |
| voltage-gated channel activity | 0.000443 | 0.009558 | 0.008059 | 14 |
| integrin binding | 0.000817 | 0.017013 | 0.014346 | 11 |
| scavenger receptor activity | 0.00098 | 0.019708 | 0.016617 | 7 |
| ion channel binding | 0.001016 | 0.019748 | 0.016651 | 11 |
| peptide hormone binding | 0.001064 | 0.020015 | 0.016877 | 6 |
| peptide binding | 0.001213 | 0.022098 | 0.018633 | 16 |
| Wnt-protein binding | 0.001332 | 0.022839 | 0.019257 | 5 |
| oxygen binding | 0.001332 | 0.022839 | 0.019257 | 5 |
| hormone activity | 0.001422 | 0.02328 | 0.019629 | 10 |
| monooxygenase activity | 0.001438 | 0.02328 | 0.019629 | 9 |
| ion channel regulator activity | 0.00153 | 0.024112 | 0.020331 | 10 |
| voltage-gated cation channel activity | 0.001767 | 0.027112 | 0.022861 | 10 |
| chemoattractant activity | 0.001861 | 0.027822 | 0.02346 | 5 |
| channel regulator activity | 0.002117 | 0.030862 | 0.026022 | 11 |
| potassium ion transmembrane transporter activity | 0.00233 | 0.03313 | 0.027935 | 10 |
| delayed rectifier potassium channel activity | 0.00251 | 0.034841 | 0.029377 | 4 |
| chemorepellent activity | 0.003086 | 0.041834 | 0.035274 | 3 |
| ammonium ion binding | 0.003638 | 0.04389 | 0.037008 | 6 |
| sodium ion transmembrane transporter activity | 0.003654 | 0.04389 | 0.037008 | 10 |
| pattern binding | 0.003689 | 0.04389 | 0.037008 | 4 |
| extracellular matrix structural constituent conferring compression resistance | 0.003689 | 0.04389 | 0.037008 | 4 |
| polysaccharide binding | 0.003689 | 0.04389 | 0.037008 | 4 |
| alpha-actinin binding | 0.003689 | 0.04389 | 0.037008 | 4 |
| adenylate cyclase binding | 0.004145 | 0.047999 | 0.040472 | 3 |
| monovalent inorganic cation transmembrane transporter activity | 0.004309 | 0.047999 | 0.040472 | 17 |
| amide binding | 0.004309 | 0.047999 | 0.040472 | 17 |
| neurotransmitter binding | 0.004364 | 0.047999 | 0.040472 | 5 |
