# Supplementary material for: Significance of tumor mutation burden combined with immune infiltrates in the progression and prognosis of ovarian cancer
Source: Cancer Cell Int. 2020 Aug 5;20:373. doi: 10.1186/s12935-020-01472-9 (PMC7405355; doi:10.1186/s12935-020-01472-9)
Supplement: Supplementary file 2 — Additional file 2: Table S2. KEGG pathway functional enrichment analysis of the differentially expressed genes. [file 12935_2020_1472_MOESM2_ESM.docx]

| Description | pvalue | p.adjust | qvalue | gene count |
| --- | --- | --- | --- | --- |
| Neuroactive ligand-receptor interaction | 5.18E-13 | 1.26E-10 | 1.06E-10 | 41 |
| Calcium signaling pathway | 2.65E-08 | 3.22E-06 | 2.72E-06 | 24 |
| Vascular smooth muscle contraction | 7.63E-08 | 6.18E-06 | 5.22E-06 | 19 |
| Dilated cardiomyopathy (DCM) | 6.33E-07 | 3.85E-05 | 3.25E-05 | 15 |
| Arrhythmogenic right ventricular cardiomyopathy (ARVC) | 1.46E-06 | 6.46E-05 | 5.46E-05 | 13 |
| Hypertrophic cardiomyopathy (HCM) | 1.59E-06 | 6.46E-05 | 5.46E-05 | 14 |
| cAMP signaling pathway | 1.15E-05 | 0.000399 | 0.000337 | 21 |
| cGMP-PKG signaling pathway | 4.46E-05 | 0.001354 | 0.001144 | 17 |
| Protein digestion and absorption | 7.63E-05 | 0.002061 | 0.001741 | 12 |
| Cell adhesion molecules (CAMs) | 0.000122 | 0.002968 | 0.002507 | 15 |
| Insulin secretion | 0.000136 | 0.002993 | 0.002529 | 11 |
| Pancreatic secretion | 0.000153 | 0.003098 | 0.002617 | 12 |
| ECM-receptor interaction | 0.000167 | 0.003122 | 0.002637 | 11 |
| Focal adhesion | 0.000383 | 0.006644 | 0.005613 | 17 |
| Renin secretion | 0.000475 | 0.007338 | 0.006198 | 9 |
| Adrenergic signaling in cardiomyocytes | 0.000483 | 0.007338 | 0.006198 | 14 |
| Tyrosine metabolism | 0.001156 | 0.016522 | 0.013956 | 6 |
| Morphine addiction | 0.00345 | 0.046581 | 0.039347 | 9 |
