# Supplementary material for: Significance of tumor mutation burden combined with immune infiltrates in the progression and prognosis of ovarian cancer
Source: Cancer Cell Int. 2020 Aug 5;20:373. doi: 10.1186/s12935-020-01472-9 (PMC7405355; doi:10.1186/s12935-020-01472-9)
Supplement: Supplementary file 3 — Additional file 3: Table S3. Univariate Cox PHR of the differentially expressed genes. [file 12935_2020_1472_MOESM3_ESM.docx]

| id | HR | HR.95L | HR.95H | pvalue |
| --- | --- | --- | --- | --- |
| CDH2 | 1.043852 | 1.021591 | 1.066597 | 9.53E-05 |
| RBMS3 | 1.220328 | 1.103702 | 1.349278 | 0.000102 |
| GDF6 | 2.074765 | 1.435367 | 2.998987 | 0.000103 |
| AARD | 1.239061 | 1.111786 | 1.380905 | 0.000106 |
| NUDT10 | 1.81255 | 1.328835 | 2.472345 | 0.000173 |
| ADAMTS8 | 1.08081 | 1.037794 | 1.12561 | 0.000177 |
| ABCC9 | 1.122616 | 1.056308 | 1.193086 | 0.000196 |
| LRCH2 | 1.528115 | 1.215076 | 1.9218 | 0.000288 |
| AMHR2 | 1.121699 | 1.052601 | 1.195334 | 0.0004 |
| CPA3 | 1.015986 | 1.00709 | 1.024961 | 0.000408 |
| KCNT2 | 2.841696 | 1.56441 | 5.161841 | 0.000605 |
| SELP | 1.074926 | 1.031388 | 1.120303 | 0.000615 |
| CYP1B1 | 1.030477 | 1.0129 | 1.048359 | 0.000626 |
| PLA2G5 | 1.129857 | 1.05331 | 1.211967 | 0.000647 |
| DDR2 | 1.038962 | 1.016233 | 1.0622 | 0.000707 |
| TCEAL7 | 1.149671 | 1.059437 | 1.247591 | 0.000825 |
| PABPC5 | 2.799704 | 1.527763 | 5.130601 | 0.000864 |
